# The secreted inhibitor of invasive cell growth CREG1 is negatively regulated by cathepsin proteases

**DOI:** 10.1007/s00018-020-03528-5

**Published:** 2020-05-08

**Authors:** Alejandro Gomez-Auli, Larissa Elisabeth Hillebrand, Daniel Christen, Sira Carolin Günther, Martin Lothar Biniossek, Christoph Peters, Oliver Schilling, Thomas Reinheckel

**Affiliations:** 1 Institute of Molecular Medicine and Cell Research, Faculty of Medicine, University of Freiburg, 79104 Freiburg, Germany; 2 Institute of Surgical Pathology, University Medical Center, Faculty of Medicine, University of Freiburg, 79106 Freiburg, Germany; 3 German Cancer Research Center (DKFZ) Heidelberg, and German Cancer Consortium (DKTK), Partner Site Freiburg, 79104 Freiburg, Germany; 4 BIOSS Centre for Biological Signalling Studies, University of Freiburg, 79104 Freiburg, Germany

**Keywords:** Breast cancer, Cathepsin, Cysteine protease, Interstitial fluid, Lysosome, Tumor microenvironment

## Abstract

Previous clinical and experimental evidence strongly supports a breast cancer-promoting function of the lysosomal protease cathepsin B. However, the cathepsin B-dependent molecular pathways are not completely understood. Here, we studied the cathepsin-mediated secretome changes in the context of the MMTV-PyMT breast cancer mouse model. Employing the cell-conditioned media from tumor-macrophage co-cultures, as well as tumor interstitial fluid obtained by a novel strategy from PyMT mice with differential cathepsin B expression, we identified an important proteolytic and lysosomal signature, highlighting the importance of this organelle and these enzymes in the tumor micro-environment. The *Cellular Repressor of E1A Stimulated Genes 1* (CREG1), a secreted endolysosomal glycoprotein, displayed reduced abundance upon over-expression of cathepsin B as well as increased abundance upon cathepsin B deletion or inhibition. Moreover, it was cleaved by cathepsin B in vitro. CREG1 reportedly could act as tumor suppressor. We show that treatment of PyMT tumor cells with recombinant CREG1 reduced proliferation, migration, and invasion; whereas, the opposite was observed with reduced CREG1 expression. This was further validated in vivo by orthotopic transplantation. Our study highlights CREG1 as a key player in tumor–stroma interaction and suggests that cathepsin B sustains malignant cell behavior by reducing the levels of the growth suppressor CREG1 in the tumor microenvironment.

**Electronic supplementary material:**

The online version of this article (10.1007/s00018-020-03528-5) contains supplementary material, which is available to authorized users.

## Introduction

Many proteolytic enzymes are mechanistically linked to the progression and metastasis of carcinomas [1]. In this regard, the eleven members of the human cysteine cathepsin protease family have been intensely studied for their clinical prognostic value, their use as prodrug activators, and for the consequences of their inhibition for tumor phenotypes [2, 3]. Although most findings validate that pharmacologic inhibition or genetic ablation of cysteine cathepsin activities reduces malignant growth, invasion, and metastasis, the identification of cathepsin substrate proteins that mechanistically link cathepsin proteases with malignant cell behavior is lagging behind [2, 4]. One reason might be that cathepsins have a high capability for complete substrate protein degradation, especially in their bona fide localization in the acidic endolysosomal compartment [2, 4, 5]. In addition, cathepsins are known to be secreted from tumor cells as well as from immune cells, with tumor-associated macrophages (TAMs) as the best-studied example [6–9]. For the latter, it is well established that macrophage-derived cathepsins promote not only tumor progression and metastasis, but also chemotherapy resistance [9, 10]. Yet, it has not been elucidated how such effects might occur. One line of thought favors that extracellular cathepsins are stabilized and active in the relatively acidic cancer micro-milieu and are, therefore, able to remodel the extracellular matrix (ECM) by degrading its constituents [2, 11, 12]. Consequently, cancer cells are thought to be able to invade the tumor stroma more easily. However, this idea does not provide a stringent explanation of the frequently observed anti-proliferative effects of cathepsin inhibition on cancer cell proliferation [2, 13]. This means that the genuine functions of active cathepsins must be either the activation of growth-promoting substrates or the inactivation of growth-suppressive proteins. The latter might be more likely to occur, because there are relatively few examples for selective activating cleavages by cathepsins, especially outside the secretory cell compartment [12].

To address those general issues of cysteine cathepsin involvement in cancer progression, we focused on cathepsin B (CTSB). This protease, often together with the closely related cathepsin Z (CTSZ; also termed cathepsin X), has been shown to promote carcinomas in a number of stringent gain- and loss-of-function studies, including human cancer cell in vitro and xenograft studies, as well as in multiple genetic mouse models of cancer [14, 15]. Furthermore, inverse correlations of CTSB expression and prognosis of cancer patients have been frequently reported [2]. In terms of tumor biology, CTSB was the first among the cysteine cathepsins shown to impair lung colony formation upon tail-vein injection of CTSB proficient cancer cells, in otherwise CTSB-deficient mice [9]. Since then, further evidence for stromal, i.e., macrophage, CTSB in tumor promotion has been accumulating [7, 8, 10, 16–18]. It also became clear that CTSB from both cancer cells and macrophages (Mɸ) cooperate in driving cancer progression [19]. Still, there is a lack of knowledge regarding the intercellular mechanisms by which CTSB mediates these effects. To address these questions and to identify CTSB-regulated proteins in the tumor microenvironment, we employed the transgenic MMTV-PyMT mouse model for metastasizing breast cancer in which we have previously performed extensive CTSB loss- and gain-of-function studies [9, 13, 16, 19–21]. We analyzed the proteome secreted by co-cultures of cancer cells and macrophages with variable genotypes of CTSB and the closely related CTSZ in vitro. These screens were complemented by proteome analysis of tumor interstitial fluid (TIF) derived from MMTV-PyMT primary breast cancers with graded CTSB and CTSZ expression levels. Both analyses indicated the abundance of the glycoprotein “Cellular Repressor of E1A Stimulated Genes 1” (CREG1) to be inversely correlated to CTSB expression levels, i.e. high CREG1 in CTSB knock-out and low CREG1 in CTSB overexpressing conditions. As CREG1 has been described as a proliferation suppressive protein, we assessed its effects in the context of the MMTV-PyMT breast cancer model with the conclusion that CREG1 is a cathepsin-controlled extracellular suppressor of invasive tumor growth.

## Materials and methods

### Animal keeping and model

Mice expressing the polyomavirus middle T oncogene under transcriptional control by the MMTV LTR promoter (FVB/N-Tg(MMTV-PyVT)634Mul/J) [20] (PyMT mice) were bred to generate, as previously reported, mice wild type for *Ctsb* (PyMT^+/0^; *Ctsb*^+/+^) [9], deficient for *Ctsb* (PyMT^+/0^; *Ctsb*^−/−^) [9], deficient for both *Ctsb* and *Ctsz* (PyMT^+/0^; *Ctsb*^−/−^; *Ctsz*^−/−^) [21], or containing the human *CTSB* gene (PyMT^+/0^; Tg(CTSB)^+/0^) [16]. Female Rag2^−/−^ γc^−/−^ lymphocyte-deficient mice [22, 23] were used for orthotopic transplantation experiments. All mice work was carried out following institutional guidelines, with ethical and legal approval by the regional council of Freiburg (Registration Numbers G14/18 and G15/23) and in accordance with the German law for animal protection as published on May 18th, 2006 with last amendment on July 28th of 2014.

### Isolation and culture of tumor cells and differentiation of murine macrophages (Mɸ)

Primary PyMT tumor cells were isolated from 14-week-old tumor-bearing mice having the distinct CTSB phenotypes, as described above, and cultured as reported previously [9]. Immortalized PyMT cell lines were generated by spontaneous immortalization of primary cells as described before [19, 24]. Bone marrow-derived Mɸ from *Ctsb*^+/+^, *Ctsb*^−/−^, and *Ctsb*^−/−^; *Ctsz*^−/−^ PyMT mice were obtained and differentiated as previously described [19]. For co-cultures, primary tumor cells were plated in a 1:1 ratio with Mɸ and cultured to near confluence. Cells were cultivated in Dulbecco’s modified Eagle’s medium (DMEM, Thermo Fisher Scientific, Waltham, MA, USA) supplemented with 10% FCS (PAN-Biotech, Aidenbach, Germany), 1% l-glutamine, and 1% penicillin/streptomycin (both Thermo Fisher Scientific) at 37 °C, in a 5% CO_2_ humified incubator.

### Collection of cell-conditioned medium

Cell-conditioned medium (CCM) was collected after 24 or 48 h for western blotting or proteomics analysis, respectively, the latter processed as previously described [25]. In short, cultures were rinsed several times with pre-warmed DMEM (Thermo Fisher Scientific) and cultivated in serum-deprived DMEM for 48 h. Subsequently, the CCM was collected and supplemented with protease inhibitors ethylenediaminetetraacetic acid (EDTA; 5.0 mM), trans-epoxysuccinyl-l-leucylamido(4-guanidino)butane (E64; 0.01 mM), and phenylmethanesulfonyl fluoride (PMSF; 1.0 mM) (all AppliChem GmbH, Darmstadt, Germany), centrifuged and filtered (0.2 µm, Acrodisc, Pall Corporation, Port Washington, NY, USA). Protein concentration was determined by the Bradford method (Bio-Rad Protein Assay, Bio-Rad Laboratories, Hercules, CA, USA).

### Collection of tumor interstitial fluid (TIF) and tumor cell lysate

Tumor interstitial fluid (TIF) of 14-week-old tumor-bearing PyMT mice was collected following the previously reported method [26]. Briefly, mice were anesthetized, mammary tumors were carefully dissected and excised. The obtained masses (1.0–2.0 g) were centrifuged (130 g) for 12 min at 4 °C in an in-house-made TIF collecting tube and supplemented with protease inhibitors (5-mM EDTA, 0.01-mM E64, PMSF 1 mM). For mass-spectrometry analysis, up to 300-μg proteins were depleted of abundant proteins using Seppro^®^ mouse spin columns (Sigma-Aldrich, St. Louis, MO, USA), as previously described [26]. Tumor lysate was obtained by mechanical dispersion and homogenization (Ultra-turrax T8, Merck, Darmstadt, Germany) in ice-cold RIPA buffer (150-mM NaCl, 50-mM Tris pH 7.5, 0.25% Sodium deoxycholate, 1% Nonidet P-40, 0.1% SDS) and cleared by centrifugation. The BCA assay (Thermo Fisher Scientific) and Bradford assay (Bio-Rad) were used for protein concentration determination.

### Quantitative secretome comparison

Prior to mass-spectrometry, samples were precipitated, resolubilized, and trypsin digested, followed by dual isotopic labeling using dimethylation with either “light” formaldehyde (20 mM CH_2_O; Sigma-Aldrich) or “heavy” formaldehyde (20 mM ^13^CD_2_O; Cambridge Isotope Laboratories, Tewksbury, MA, USA) plus sodium cyanoborohydride (20-mM NaBH_3_CN; Sigma-Aldrich) as described previously [25, 27] to compare the different conditions. To reduce systematic labeling errors due to label preference, a label swap was done between some experimental replicates. Samples were then mixed in a 1:1 ratio. CCM samples and the first sample (exp1) of the TIF experiments were desalted using a C18 solid-phase extraction column (Grace-Vydac, Grace, Columbia, MD, USA) followed by fractionation with strong cation-exchange chromatography using a polysulfoethyl column (PolyLC, Columbia, MD, USA) [25]. Eluted peptides were collected in 5–9 fractions, desalted using in-house-packed 2-layer C18 STAGE-tips (Empore, 3 M, Maplewood, MN, USA) [28]. For TIF experiments 2–6, a high pH reversed-phase fractionation followed by fractions concatenation was employed [29, 30]. Twelve fractions were collected together with a pre-fractionation whole sample.

### Liquid chromatography–tandem mass spectrometry (LC–MS/MS)

CCM samples were measured on a QSTAR Elite (AB Sciex, Darmstadt, Germany) coupled to a Dionex Ultimate 3000 pump (Thermo Fisher Scientific) as described previously [31]. TIF samples were analyzed on a Q Exactive Plus (Thermo Fisher Scientific) coupled to an EASY-nLC 1000 liquid chromatograph (Thermo Fisher Scientific) as described before [32]. Mass spectrometers were operated in data-dependent mode for MS and MS/MS.

### Processing of mass spectrometry data

Obtained files from the QSTAR analysis (wiff) were converted to mzXML using the mzWiff converter (v.4.3.1, Seattle Proteome Center) using centroiding at MS and MS/MS level, deisotoping, and determining precursor charge, for peptide and protein identification and to mzML for quantitation using the ProteoWizard msconvert (v.3.0.10385) [33]. RAW files obtained from the Q Exactive analysis were converted into mzML using the ProteoWizard converter. A revised UniProt mice database without isoforms (Downloaded May 2018), including 16966 entries plus the contaminants database present in MaxQuant was used [34]. A decoy database was then generated using the DecoyPyrat tool [35] and interleaved.

Peptide and protein identification was carried out using Comet (v.2018.01rev.1) [36], X! Tandem (v.2013.06.15.1) [37] and MSGF+ (v.2018.04.09) [38] doing two static searches, one for the light and one for the heavy formaldehyde modification, and using fixed cysteine carbamidomethylation and variable oxidation of methionine as modifications with each search engine. For QSTAR files, a 0.15-Da fragment monoisotopic mass error and plus 200-, minus 100-ppm parent monoisotopic mass error in X! Tandem or a precursor mass tolerance of 100 ppm in Comet and MSGF+ were used. For Q Exactive files, a 20-ppm fragment monoisotopic mass error and 10-ppm parent monoisotopic mass error in X! Tandem or a precursor mass tolerance of 10 ppm in Comet and MSGF+ were used. No missed cleavages were allowed (Comet and X! Tandem).

Search results were analyzed with PeptideProphet (Part of the Trans-Proteomic Pipeline TPP v.5.1) [39, 40] combined using iProphet [41] and protein inference was done with ProteinProphet (both part of the TPP v.5.1) [42]. A reported minimum probability was chosen to achieve a 1% FDR at both peptide and protein levels. Peptide abundance was calculated using the FeatureFinderMultiplex tool from OpenMS (v.2.3) [43–45]. Peptide abundance features were mapped (IDMapper) to the identified peptides (iProphet) followed by IDConflictResolver and MultiplexResolver in OpenMS (v.2.3). Peptides and proteins with their corresponding abundances were assembled in R (v3.6.1, R Foundation for Statistical Computing, Vienna, Austria) as follows. Peptide ratios were normalized using median centering for CCM samples and variance stabilization normalization [46, 47] for TIF samples. In both cases, only peptides without missed cleavages were used. Protein ratios were assembled by median summarization using the peptide and protein groups information obtained from ProteinProphet using in-house-developed R-scripts, expressing the ratios as the (log_2_) of co-cultures of PyMT wild-type cells with Mɸ *Ctsb*^−/−^; *Ctsz*^−/−^ over co-cultures with wild-type PyMT cells and wild-type Mɸ or as the (log_2_) of PyMT^+/0^; *Ctsb*^−/−^; *Ctsz*^−/−^ over PyMT^+/0^; *Ctsb*^+/+^; *Ctsz*^+/+^ TIF secretomes.

### Immunoblotting

Protein samples from CCM, tissue lysates, or TIF (10–80 μg) were used for western blot analysis. Protein samples were subjected to SDS-PAGE and transferred via a semi-dry system (Bio-Rad) to a polyvinylidene fluoride membrane (Amersham GE Healthcare, Buckinghamshire, UK). After blocking of membranes with 4% non-fat milk in PBS-Tween, they were incubated with primary antibodies goat anti-mouse CREG1 (R&D systems, Minneapolis, MN, USA; AF1697), goat anti-mouse CTSB (R&D systems; BAF965), goat anti-human CTSB (R&D systems; AF953), goat anti-mouse CTSZ (R&D systems; BAF1033), or mouse anti-alpha-tubulin (Sigma-Aldrich; T9026) overnight at 4 °C. Subsequently, membranes were washed and probed with the corresponding secondary antibodies rabbit anti-goat POD (Sigma-Aldrich; A5420) or goat anti-mouse POD (Sigma-Aldrich; A0168) for 1 h at room temperature. After washing the membranes, they were developed using a Pierce West Pico/Femto Chemiluminescent substrate (Thermo Fisher Scientific) and imaged with a Fusion SL Detection System (Vilber Lourmat, Eberhardzell, Germany).

### Immunohistochemistry

Harvested tumors were paraffin-embedded, processed, and blocked for unspecific antibody staining using rabbit serum (Vectastain ABC HRP kit, Vector Laboratories, Burlingame, CA, USA). Subsequently, tissue sections were stained with the primary antibody goat anti-mouse CREG1 (R&D Systems; AF1697) overnight in a humidified chamber at 4 °C. After rinsing slides in PBS-Tween, they were probed with the secondary antibody anti-goat IgG (Vectastain ABC HRP kit, Vector Laboratories) for 45 min in a humidified chamber at room temperature. Subsequently, ABC complex solution (Vectastain ABC HRP kit, Vector Laboratories) was applied to increase sensitivity for 45 min in a humidified chamber at room temperature. Lastly, a 3,3′-diaminobenzidine (DAB) substrate solution (Sigma-Aldrich) was added and the reaction was stopped by ddH_2_O. Counterstaining was achieved with Mayer’s hemalum solution (VWR, Radnor, PA, USA). Afterward, slides were dried and mounted with Aquatex (Merck Millipore, Burlington, MA, USA). Tissue sections were imaged using an Axioskop2/AxioCam microscope (Carl Zeiss, Jena, Germany) and analyzed using AxioVision software (Carl Zeiss) and Fiji/ImageJ (NIH, Bethesda, MD, USA).

### Cysteine CTSB inhibition and induction

Cysteine CTSB protease was inhibited by addition of E64d (10 µM) or CA-074 (10 µM) (both Bachem, Bubendorf, Switzerland), using DMSO as solvent control, as reported [24]. Human CTSB induction in CTSB-deficient PyMT cells was achieved by a doxycycline-inducible system based on the pTRIPZ lentiviral vector (Thermo Fisher Scientific) as described previously [19].

### Quantitative real-time PCR

RNA was isolated from tumor cells/Mɸ co-cultures employing the RNeasy Mini Kit (Qiagen, Hilden, Germany) and transcribed to cDNA using the iSCRIPT cDNA synthesis system (Bio-Rad). CREG1 was detected by qRT-PCR with the following primer pair: CREG1: fw 5′TCAATCAGTGACGGTCCTCC 3′, rev 5′GTCAGCGTAGCCTCTGGATT 3′; and normalized to β-actin using the following primer pair: β-actin: fw 5′ACCCAGGCATTGCTGACAGG 3′, rev 5′GGACAGTGAGGCCAGGATGG 3′. Samples were measured on a Bio-Rad iQ5 or CFX96, Real-Time Systems (Bio-Rad) and analyzed using a relative quantification strategy. Statistics were done using dCT values and data are presented as fold change over control.

### Cell growth, migration, and invasion

For real-time monitoring of cell growth, migration, and invasion, the RTCA device, xCELLigence RTCA DP (Acea Biosciences, San Diego, CA, USA) was employed. For the assessment of cell growth, tumor cells were seeded in triplicates into E-plates 16^®^ at a concentration of 8000 cells per well in DMEM supplemented with 3% FCS (PAN-Biotech). Impedance was measured for up to 48 h every 15 min. For the analysis of the influence of extracellular CREG1 on cell growth after overnight incubation/monitoring, murine recombinant CREG1 (R&D systems) was added to a final concentration of 400 nM [48] or an equal volume of PBS as control. Cell growth was monitored for at least 24 more hours. CIM-plates 16^®^ were used for the analysis of migration and invasion. For migration, the lower chamber, containing 150 µl of 3% FCS in DMEM, was coupled to the upper chamber, in which a cell concentration of 60,000 cells in serum-deprived medium was added in triplicates. For invasion, the upper chambers of the CIM-plates 16^®^ were coated with 30 µl of Cultrex^®^ (Trevigen, Gaithersburg, MD, USA) in a 1:22.5 dilution. After solidification, 60,000 cells per well were added in triplicates to the upper chamber of the CIM plate. For migration and invasion, the impedance was measured for at least 24 h in 15-min intervals. Extracellular CREG1 influence on migration and invasion was assessed by adding murine recombinant CREG1 (R&D systems) to the upper chamber in a final concentration of 400 nM as described above.

### Gap-closure assay

Wild-type tumor cells (PyMT), or wild-type tumor cells harboring shControl or shCreg1 were seeded into both openings of a silicon insert (ibidi GmbH, Martinsried, Germany), on µ-Slide 8-well ibidi plates (ibidi), at a concentration of 35,000 cells/opening. Cells were grown overnight. The insert was removed, creating a defined 500-µm gap. Wells were washed once with PBS and media were replaced to 3% FCS DMEM with or without 400-nM recombinant CREG1. Subsequently, three–four fields per well in triplicates were imaged with a JuLI™ Stage live-cell imaging camera (NanoEntek, Seoul, Korea) for 24 h in 45-min intervals. Images were analyzed using Fiji/ImageJ software (NIH) with the Montpellier Ressources Imagerie (MRI) Wound healing tool (https://github.com/MontpellierRessourcesImagerie/imagej_macros_and_scripts/wiki/Wound-Healing-Tool), and summarized in R.

### RNAi-mediated CREG1 silencing

To generate stable cell lines of tumor cells (PyMT) or Mɸ with reduced mRNA expression of CREG1, designed short hairpin (sh) RNA plasmids from The RNAi Consortium (TRC, Broad Institute) were obtained and used following the manufacturer’s recommendations (Thermo Fisher Scientific Open Biosystems) and TRC laboratory protocols. The shRNA constructs for CREG1 TRC91-93: TRCN00000993XX (XX = 90–93), shCreg1 (TRC93): TRCN0000099393 (ATTCCTACAGTAGACAGTCTG) and non-target shRNA control plasmid DNA (SCR: SHC016-1EA; Sigma-Aldrich) which are cloned into the pLKO.1 TRC lentiviral vector were employed. Stable cell lines were generated using the pMISSION system (Sigma-Aldrich) and selected using Puromycine (5 µg/ml) for 10 days (Sigma-Aldrich) as previously reported [19].

### Three-dimensional spheroid sprouting assay

Tumor cells were suspended in DMEM with 0.24% (w/v) methylcellulose (Sigma-Aldrich) solution and cultured as hanging droplets (500 cells per drop) overnight to generate spheroids as described before [19]. Subsequently, spheroids were embedded in a collagen I matrix (Becton Dickinson, Franklin Lakes, NJ, U.S.) with 0.6% methylcellulose and with or without the addition of Mɸ shControl or shCreg1 in a 1:1 ratio. After solidification, medium was added and the spheroids were cultured for 24–48 h, after which phase-contrast images of spheroids were acquired with a Zeiss Axio Observer Z1 (Carl Zeiss) or a Keyence BZ-9000 microscope (Keyence, Osaka, Japan). Invasiveness and collective cell migration of spheroids were measured by analyzing the number and length of invasive strands with Fiji/ImageJ software (NIH). To analyze the impact of extracellular CREG1 on spheroid sprouting of wild-type tumor cells, murine recombinant CREG1 was added (400 nM) together with DMEM medium on top of the collagen matrix.

### Orthotopic transplantation assay

Immortalized PyMT cells harboring a shControl or shCreg1 construct were resuspended in 25-µl DMEM (Thermo Fisher Scientific) containing 2.5 × 10^5^ cells, mixed with an equal volume of Cultrex^®^ (Trevigen), and transplanted into the fourth mammary gland of adult female Rag2^−/−^ γc^−/−^ lymphocyte-deficient mice via a 5-mm lateral incision. Animals were followed up weekly by palpation for 4 weeks. After 4 weeks, mice were euthanized, tumors were harvested, and analyzed. Volumes were calculated following an ellipsoid.

### Cleavage assay

To analyze in vitro processing of CREG1 by CTSB and CTSZ, a cleavage assay was carried out. Mouse recombinant CTSB (200 ng) and/or mouse recombinant CTSZ (200 ng) were activated in sodium acetate buffer (100-mM sodium acetate, 2-mM EDTA, 2-mM cysteine, pH 5.0) or phosphate citrate buffer (100-mM citric acid/disodium phosphate, 2-mM cysteine, pH 5.0) containing 5-mM DTT, incubating for 15 min at room temperature, and then added to 2-µg recombinant murine CREG1 (all R&D Systems) in sodium acetate buffer either at pH 5.0 or phosphate citrate buffer at pH 6.6. The mixtures were incubated for 0, 6 (only for pH 5.0), and 24 h at 37 °C. To stop the reaction, Laemmli sample buffer containing E64 (100 µM, AppliChem GmbH) was added to the mix and heated for 5 min at 95 °C. Samples were subjected to SDS-PAGE, followed by gel fixation in 40% ethanol, 10% acetic acid and stained using Coomassie blue G250 for 24 h. Gels were destained using 20% methanol and imaged.

### N-terminal sequencing (Edman degradation)

For N-terminal sequencing, samples were processed as previously described for the cleavage assay but prior to SDS-PAGE, samples were reduced with DTT followed by alkylation with iodoacetamide. Afterward, samples were blotted to PVDF membranes (Amersham GE Healthcare) in a semi-dry transfer system (Bio-Rad Trans-blot turbo) using sodium borate buffer (50 mM, 20% methanol, 0.1% SDS, pH 9.0). Subsequently, membranes were stained with Coomassie blue (0.1% CBB R250, 10% acetic acid, 40% methanol) followed by destaining (40% methanol, 10% acetic acid). Membranes were dried and sent for N-terminal sequencing analysis to Proteome Factory AG (Berlin, Germany). Five steps per reaction were obtained.

### Data and statistical analysis of mass spectrometry data

Identified proteins were batch queried to UniProt [49] to obtain UniProt and GO annotation. The prediction servers SecretomeP 2.0 [50] and SignalP 5.0 [51] were used to obtain information about potential non-classical secretion. Additionally, the protease and protease inhibitors MEROPS database [52] and Mouse Lysosome Gene Database (mLGDB) [53] were downloaded (June 2019) and matched. Cellular compartment localization was then obtained by in-house-developed scripts using the queried information. Only proteins consistently identified in at least three experiments were included for downstream analysis. The overlap of the identified proteins between experiments was determined in R and visualized using the UpSetR R-package [54]. Quantitation data were analyzed by fitting a linear model using the R/Bioconductor package limma [55, 56] as before [26]. For the CCM secretome, proteins were considered to have an altered abundance if the limma moderated *p* value was ≤ 0.05 and the fold change (FC) was 30% more or less. For the TIF analysis, only proteins classified as being secreted and/or lysosomal were employed. TIF proteins were considered to have an altered abundance if the limma moderated *p* value was ≤ 0.025 and the FC had a more/less 50% change.

### Data presentation and statistics

For statistical analysis comparing the difference between means of two groups, the two-tailed Student’s *t* test was used. Multiple group comparisons were done by analysis of variance (ANOVA) followed by a post hoc Tukey range test. xCELLigence assays were analyzed by fitting a linear model of the slopes, followed by a likelihood ratio test of the fitted model. For closure of the gap experiments, a linear mixed-effects model with time as a random variable was employed followed by likelihood ratio test of the fitted models. Statistical analysis and graphics were done in R (R Foundation for Statistical Computing) using RStudio as an IDE (RStudio: Integrated Development for R. RStudio, Inc., Boston, MA) and OriginPro 2016 (OriginLab, Northampton, MA, USA).

## Results

### CTSB and CTSZ influence the secretome upon tumor cell–macrophage interaction

**Fig. 1:**
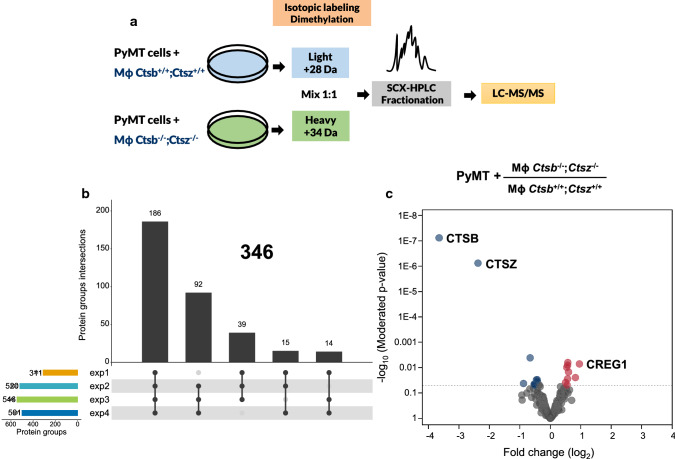
Influence of CTSB and CTSZ on the secretome of tumor cell–macrophage interactions. **a** Wild-type PyMT cells were co-cultured with macrophages wild type or lacking both *Ctsb* and *Ctsz*. After cultivation in serum-free media, the CCM was collected, labeled using dimethylation, fractionated using SCX–HPLC, and subject to LC–MS/MS. **b** 346 proteins were consistently identified in 3 out of 4 experiments. **c** Using these proteins, a linear model was fitted and proteins with a change of more/less than 30% and a limma *p* value ≤ 0.05 were considered to have an altered abundance. The vast majority of proteins show minor quantitative differences. *exp* experiment, *SCX* strong cation-exchange chromatography

First, we asked how lysosomal cathepsins might affect the composition of the secreted proteome (i.e., the secretome) of PyMT breast cancers. We considered cancer cells as well as TAMs as important sources for secreted proteins. Therefore, we performed a quantitative secretome comparison using differential isotope labeling of the cell-conditioned medium (CCM) of co-cultures comprising PyMT breast cancer cells and Mɸ either wild type or double knock-out for CTSB and CTSZ (Mɸ *Ctsb*^−/−^; *Ctsz*^−/−^) (Fig. 1a). In four independent biological experiments, on average, 470 proteins were identified and quantified in the CCM (exp1: 311, exp2: 520, exp3: 546, exp4: 501) (Fig. 1b). From these, only proteins consistently identified in at least three experiments were used for downstream statistical analysis (*n* = 346) (Fig. 1b; Supplementary File 1).

Gene ontology annotation obtained from UniProt [49] revealed a distinct proteolytic and lysosomal signature in the secreted proteins. To have a comprehensive view of these signatures first, the current MEROPS database of proteases and protease inhibitors [52] was downloaded and matched to the identified proteins. Proteases and protease inhibitors accounted for 16.5% of the consistently identified proteins (37 annotated proteases, 20 annotated protease inhibitors), further confirming our previous observation when analyzing the interstitial fluid of breast tumors from mice of the same model [26]. In addition, to widen the assessment of the identified lysosomal proteins, the Mouse Lysosomal Gene Database (mLGDB) [53] was matched. Notwithstanding, 47 of the 346 analyzed proteins were classified as lysosomal proteins (13.6%) (Supplementary File 1).

Differential isotopic labeling of tryptic peptides by “light” or “heavy” formaldehyde allowed for the quantitative comparison of the CCM proteome. To identify differentially regulated proteins, protein ratios were quantile normalized and analyzed by a linear modeling strategy, as described previously [26, 55, 56]. Using this strategy, 19 proteins were found to have significantly altered abundance (Fig. 1c; Table 1). Strikingly, the co-cultures of PyMT breast cancer cells expressing CTSB and CTSZ and Mɸ being double deficient for these proteases revealed a reduction of those enzymes to levels of 7.97% (CTSB, FC log_2_(KO/wt)-3.65) and 19.3% (FC log_2_(KO/wt)-2.37) compared to co-cultures in which cancer cells and Mɸ were wild type for both CTSB and CTSZ. This result is in concordance with previous findings showing that Mɸ are the main source of cathepsin secretion into the tumor microenvironment [7, 10]. Eight additional proteins with decreased abundance in the absence of both cathepsin proteases from Mɸ were identified, including the latent-transforming growth factor beta-binding protein 2 (O08999), thioredoxin (P10639), the protease inhibitor antileukoproteinase (P97430), and the insulin-like growth factor-binding proteins 2 (P47877). On the other hand, we found nine proteins with consistently increased levels when CTSB and CTSZ were deleted in Mɸ. These included several lysosomal proteins such as legumain (O89017), beta-mannosidase (Q8K2I4), cathepsin L1 (P06797), dipeptidyl peptidase 2 (Q9ET22), and the “Cellular Repressor of E1A Stimulated Genes 1” (CREG1; O88668) (Fig. 1c), studied in more detail in this report.

**Table 1:** Proteins with altered abundance in co-cultures with Mɸ lacking *Ctsb* and *Ctsz*

UniProt	Protein name	Mean	CI 95%		*p* value
P10605	Cathepsin B	− 3.65	− 4.08	− 3.22	7.53E−08
Q9WUU7	Cathepsin Z	− 2.37	− 2.75	− 1.99	7.53E−07
Q9JI91	Alpha-actinin-2	− 0.88	− 1.71	− 0.04	0.0423
P10639	Thioredoxin	− 0.66	− 1.04	− 0.28	0.0041
P47877	Insulin-like growth factor-binding protein 2	− 0.52	− 1.03	− 0.02	0.0446
P60843	Eukaryotic initiation factor 4A-I	− 0.52	− 1.04	− 0.01	0.0470
Q8BSU2	C-X-C motif chemokine 16	− 0.51	− 1.01	0.00	0.0493
O08999	Latent-transforming growth factor beta-binding protein 2	− 0.45	− 0.85	− 0.06	3.03E−02
Q61581	Insulin-like growth factor-binding protein 7	− 0.43	− 0.80	− 0.05	0.0297
P97430	Antileukoproteinase	− 0.42	− 0.80	− 0.04	0.0348
Q8BG07	Phospholipase D4	0.50	0.04	0.97	0.0385
P06797	Cathepsin L1	0.54	0.17	0.91	0.0097
Q8CIE6	Coatomer subunit alpha	0.55	0.01	1.10	0.0479
O08553	Dihydropyrimidinase-related protein 2	0.57	0.20	0.94	7.91E−03
Q9ET22	Dipeptidyl peptidase 2	0.57	0.08	1.05	0.0276
O89017	Legumain	0.58	0.22	0.93	0.0063
Q8K2I4	Beta-mannosidase	0.60	0.16	1.04	0.0148
O70456	14-3-3 protein sigma	0.83	0.14	1.51	0.0247
O88668	Protein CREG1	0.96	0.36	1.555	0.0071

Mean fold-change ratios (log_2_) of co-cultures of PyMT wild-type cells with Mɸ *Ctsb*^−/−^; *Ctsz*^−/−^ over PyMT wild-type cells with Mɸ *Ctsb*^+/+^; *Ctsz*^+/+^

*CI 95%* 95% confidence interval

### CTSB influences the breast cancer secretome in vivo

**Fig. 2:**
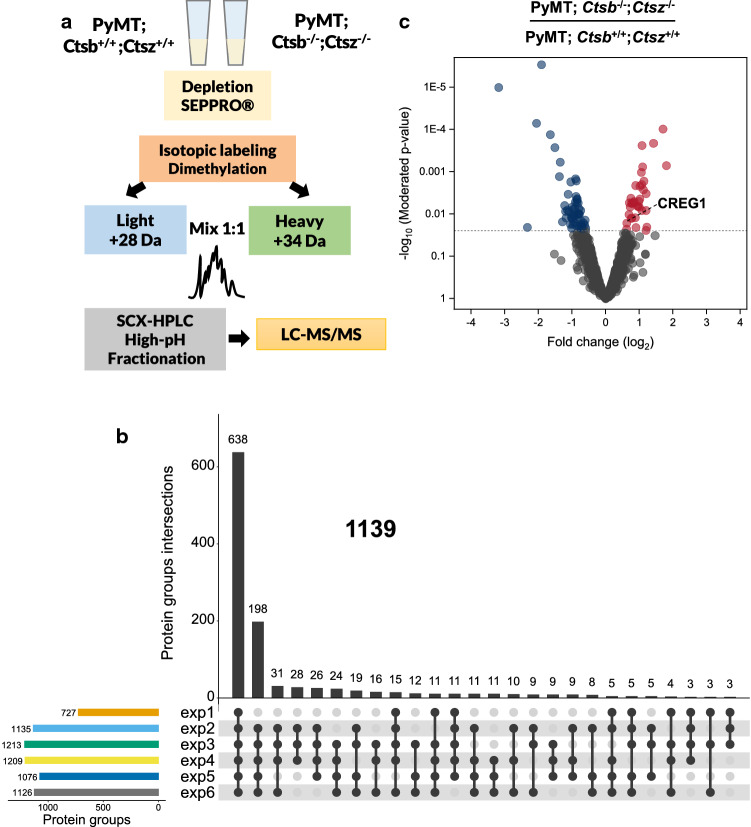
Influence of CTSB and CTSZ on the tumor interstitial fluid composition. **a** Tumors from PyMT^+/0^; *Ctsb*^+/+^; *Ctsz*^+/+^ and PyMT^+/0^; *Ctsb*^−/−^; *Ctsz*^−/−^ were carefully dissected and subject to low-speed centrifugation and the TIF was collected. The TIF was depleted of highly abundant proteins and labeled by dimethylation. Samples were either fractionated by SCX (exp1) or by high pH reversed-phase fractionation followed by fraction concatenation (exp2–6) and subject to LC–MS/MS. **b** Proteins consistently identified in 3 out of 6 experiments and classified as being secreted and/or lysosomal were considered (1139). Only up to three common protein interactions are shown. **c** A linear model was fitted and proteins with a fold change of more/less than 50% and a limma *p* value ≤ 0.025 were considered to have an altered abundance. The majority of proteins show minor quantitative differences. *exp* experiment, *SCX* strong cation-exchange chromatography

To complement the cell culture-based secretome exploration with an in vivo approach, the tumor interstitial fluid (TIF) from 14-week-old tumor-bearing PyMT mice, wild type or double knock-out for CTSB and CTSZ, was compared. Collection and preparation of the TIF were performed as previously described by our group [26], but employing a binary differential isotope labeling method for quantitative proteome comparison (Fig. 2a). Only for the first experiment, a pre-fractionation employing strong-cation exchange (SCX) was used; whereas for the remaining samples, a high pH reversed-phase fractionation followed by fractions concatenation was employed [29, 30]. With the pre-fractionation and concatenation strategy changes, the average of identified and quantified proteins per experiment increased from 1299 (exp1) to an average of 2415 (exp2–exp6) (Supplementary File 3). Of these, only proteins identified in at least 3 experiments were considered for downstream analysis, summing 2357 proteins (Supplementary File 3). As with the co-culture CCM comparison, the UniProt database was queried for detailed annotation of the identified proteins. Proteins classified as secreted accounted for 1139 (48.3% of analyzed proteome) (Fig. 2b), which is similar or better compared to current secretome studies [57–60]. By query of the MEROPS peptidase database [52], we found that 7.85% of the identified proteins were annotated as proteases (136) or protease inhibitors (49), a result further exemplifying the high abundance of proteases and their inhibitors at the tumor–stroma interaction sites. In addition, using the mLGDB database [53] together with UniProt annotation, we found about 6% of TIF proteins annotated to be of lysosomal origin (total 141 proteins; Supplementary File 3).

Using binary differential isotope labeling as described for the CCM analysis (Fig. 1), 92 TIF proteins were found to have an altered abundance due to the lack of both CTSB and CTSZ (Fig. 2c; Supplementary File 3). From these, 57 proteins had a decreased abundance including haptoglobin (Q61646), fibulin-5 (Q9WVH9), as well as proteins with reported involvement in cancer like SPARC-like protein 1 (P70663) [61], osteopontin (P10923) [62], and annotated proteases like serine protease HTRA3 (Q9D236), and the protease inhibitor antileukoproteinase (P97430). In contrast, 35 proteins were found to have an increased abundance upon protease deficiency. These included galectin-7 (O54974), the epithelial cell adhesion molecule (EpCAM; Q99JW5), *N*-acetylglucosamine-6-sulfatase (Q8BFR4), glutathione peroxidase 3 (P46412), and proteases like dipeptidyl peptidase 2 (Q9ET22). Remarkably, as in our cell culture studies, we also found the glycoprotein CREG1 with an increased abundance in cathepsin double knock-outs (Fig. 2c).

### Inverse levels of CTSB and CREG1 due to post-translational processing

**Fig. 3:**
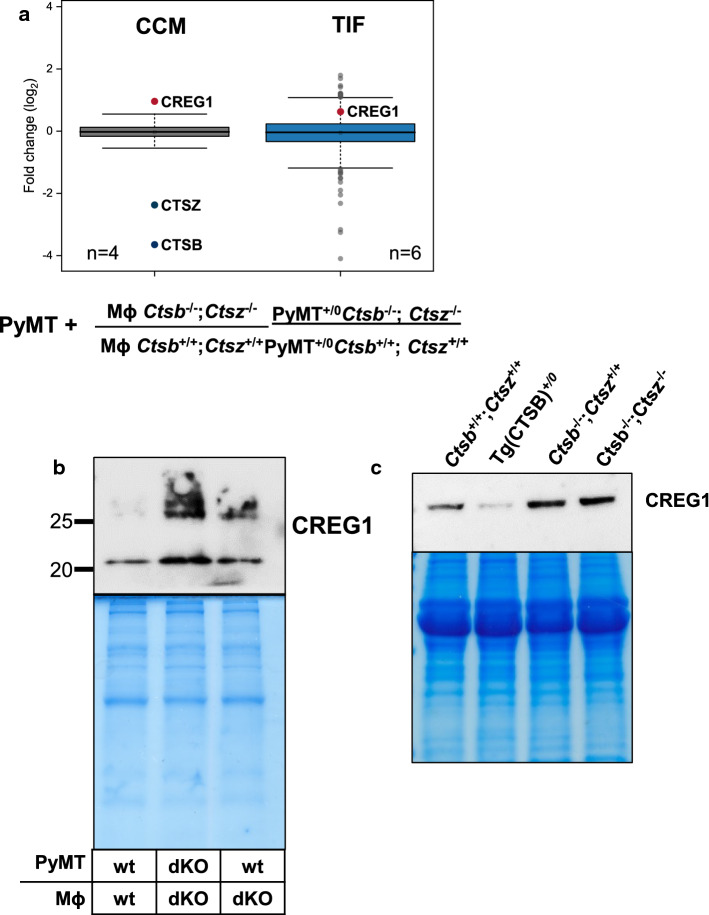
CREG1 changes upon *Ctsb* expression. **a** CREG1 abundance is increased in both co-cultures having Mϕ *Ctsb*^−/−^; *Ctsz*^−/−^, as well as in the TIF of PyMT^+/0^; *Ctsb*^−/−^; *Ctsz*^−/−^ mice in MS proteomics studies. **b** Increased abundance is also observed by western blot using the CCM of co-cultures of tumor cell/macrophage deficient of both *Ctsb* and *Ctsz* and in co-cultures of wild-type tumor cells with Mϕ *Ctsb*^−/−^; *Ctsz*^−/−^. **c** In the TIF, the abundance of CREG1 changes upon *Ctsb* expression. CREG1 is increased in the TIF of PyMT^+/0^; *Ctsb*^−/−^; *Ctsz*^+/+^ mice, as well as in PyMT^+/0^; *Ctsb*^−/−^; *Ctsz*^−/−^ mice, but decreased in the TIF with the transgenic expression of human CTSB (Tg(CTSB)^+/0^). *CCM* cell-conditioned media, *TIF* tumor interstitial fluid, *Mϕ* macrophage, *wt* wild type, *dKO* deficient from both *Ctsb* and *Ctsz*

By comparing the results of the CCM from PyMT cell/macrophage co-cultures and the TIF analysis, six proteins were identified with concordant alterations in abundance due to cathepsin deficiency (Table 2). CREG1 stands out of those hits, not only because it was found to be differentially altered in both comparisons (Fig. 3a), but also because CREG1 has been previously shown to suppress cell proliferation and to promote cell differentiation [63, 64]. Therefore, increased CREG1 levels in tumor-bearing CTSB or CTSB/CTSZ knock-out mice could mediate the reduction in tumor proliferation and invasive growth that has been consistently reported for such models [9, 13, 21, 65]. In consequence, we further explored this hypothesis in more detail.

**Table 2:** Proteins with congruent abundance change due to the absence of *Ctsb* and *Ctsz*

UniProt	Protein name	CCM PyMT + Mɸ; *Ctsb*^−/−^; *Ctsz*^−/−^				Interstitial fluid PyMT; *Ctsb*^−/−^; *Ctsz*^−/−^			
		Mean	CI 95%		*p* value	Mean	CI 95%		*p* value
P10605	Cathepsin B	− 3.65	− 4.08	− 3.22	7.53E−08	n.d.			
Q9WUU7	Cathepsin Z	− 2.37	− 2.75	− 1.99	7.53E−07	n.d.			
P47877	Insulin-like growth factor-binding protein 2	− 0.52	− 1.03	− 0.02	0.0446	− 0.81	− 1.34	− 0.28	0.0055
P97430	Antileukoproteinase	− 0.42	− 0.80	− 0.04	0.0348	− 1.09	− 1.85	− 0.33	0.0089
Q9ET22	Dipeptidyl peptidase 2	0.57	0.08	1.05	0.0276	1.08	0.61	1.55	0.0002
O88668	Protein CREG1	0.96	0.36	1.56	0.0071	0.62	0.13	1.12	0.0165

Mean fold change ratios (log_2_) of co-cultures of PyMT wild-type cells with Mɸ *Ctsb*^−/−^; *Ctsz*^−/−^ over PyMT wild-type cells with Mɸ *Ctsb*^+/+^; *Ctsz*^+/+^ and of TIF from PyMT^+/0^; *Ctsb*^−/−^; *Ctsz*^−/−^ mice over PyMT^+/0^; *Ctsb*^+/+^; *Ctsz*^+/+^

*CI 95%* 95% confidence interval, *n.d.* No confident detection in the PyMT^+/0^; *Ctsb*^−/−^; *Ctsz*^−/−^

**Fig. 4:**
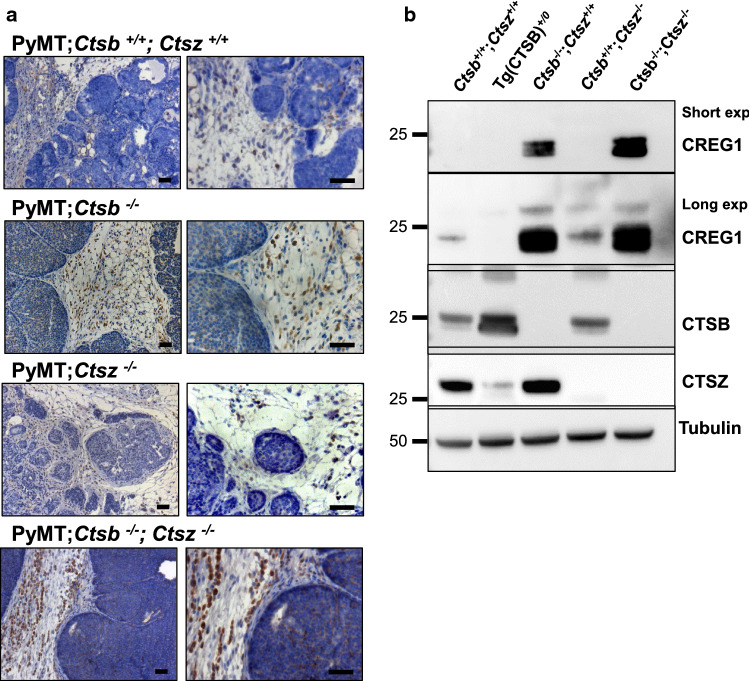
CREG1 abundance in breast cancer tissue sections of the MMTV-PyMT model. **a** Immunostaining of CREG1 in breast tumor sections of PyMT^+/0^; *Ctsb*^−/−^; *Ctsz*^−/−^ tumors shows stronger staining when compared to wild-type PyMT tumors, especially in stromal cells. A milder effect is seen in tumors from PyMT^+/0^; *Ctsb*^−/−^; *Ctsz*^+/+^ mice. No effect was observed in mice deficient for *Ctsz*. **b** Lysis of whole tumors shows an important increased abundance of CREG1 in tumors from PyMT^+/0^; *Ctsb*^−/−^; *Ctsz*^+/+^ and PyMT^+/0^; *Ctsb*^−/−^; *Ctsz*^−/−^, apparent at short exposure (upper lane). After long-exposure CREG1 is observed in tumors from wild-type mice (middle lane). *Exp* exposure

**Fig. 5:**
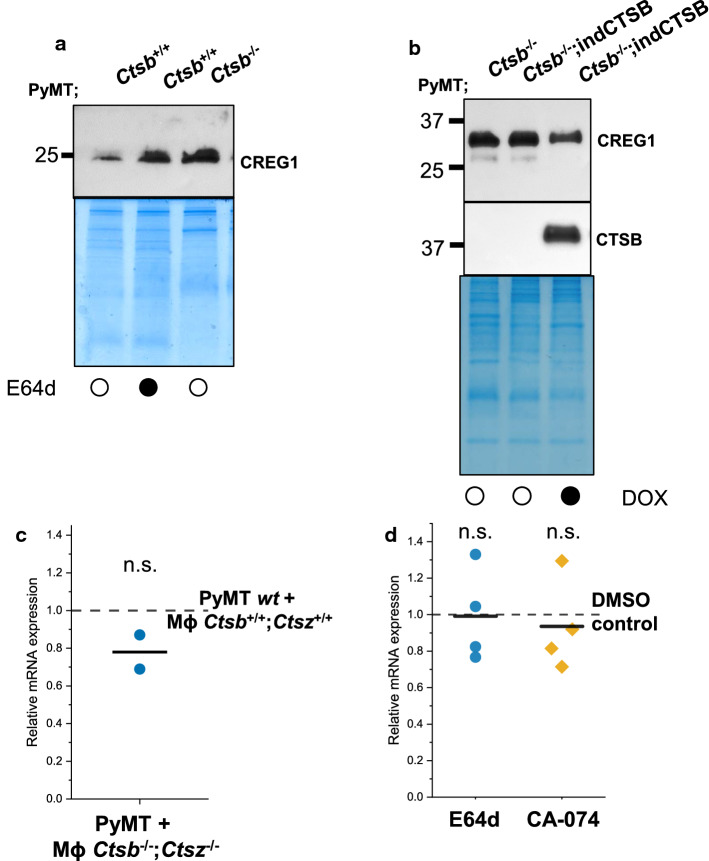
CREG1 abundance is post-translationally modulated by CTSB inhibition and CTSB induction. **a** The abundance of CREG1 is increased in PyMT cells treated with the broad spectrum CTSB inhibitor E64d (10 µM). **b** CREG1 abundance can be reduced upon doxycycline induction (1 µM) of CTSB in a PyMT *Ctsb*^−/−^ cell line carrying a CTSB doxycycline-inducible vector. **c** No significant changes of CREG1 at the mRNA level (qRT-PCR) were observed in tumor-cell co-cultures carrying Mϕ lacking both *Ctsb* and *Ctsz* compared to wild-type Mϕ (dashed line). **d** Inhibition of CTSB in PyMT cells with E64d (10 µM) or with CA-074 (10 µM) lead to no significant changes of CREG1 at the mRNA level compared to control (dashed line). *n.s.* non-significant change, *Mϕ* macrophages, *indCTSB* doxycycline-inducible CTSB, *DOX* doxycycline, *CTSB* human CTSB

CREG1 is a secreted glycoprotein as well as a bona fide lysosomal protein [66]. Therefore, we analyzed levels of secreted and intracellular (lysosomal) CREG1 in dependence of the cathepsin protease genotype of cells and breast cancer tissue (Figs. 3, 4, 5). First, the CREG1 levels in CCM of co-cultures of wild-type PyMT cells plus wild-type Mɸ were compared to co-cultures in which both cell types lacked *Ctsb* and *Ctsz*, as well as with co-cultures in which only the Mɸ lacked both cathepsins. In this experiment, the highest CREG1 abundance was observed when both tumor cells and Mɸ lacked the proteases (Fig. 3b). Western blots of CREG1 in TIF corroborated the proteome study, as an increased abundance of CREG1 was detected in TIF of mice lacking *Ctsb* alone or lacking both *Ctsb* and *Ctsz* (Fig. 3c). Conversely, in a gain-of-function approach utilizing transgenic mice overexpressing human CTSB in breast cancer [16, 19], we observed a reduced abundance of CREG1 in TIF (Fig. 3c). Next, immunohistochemistry for CREG1 in PyMT tumor sections revealed increased CREG1 staining in specimens derived either from *Ctsb/Ctsz* double knock-out cancers or from *Ctsb* single knock-outs but not in the *Ctsz* single knock-out (Fig. 4a). This was supported by CREG1 immunoblots of protein extracts from whole tumors (Fig. 4b). Again, a reduced abundance of CREG1 was evident in tumor lysates from mice with transgenic overexpression of human CTSB (long exposure, Fig. 4b).

**Fig. 6:**
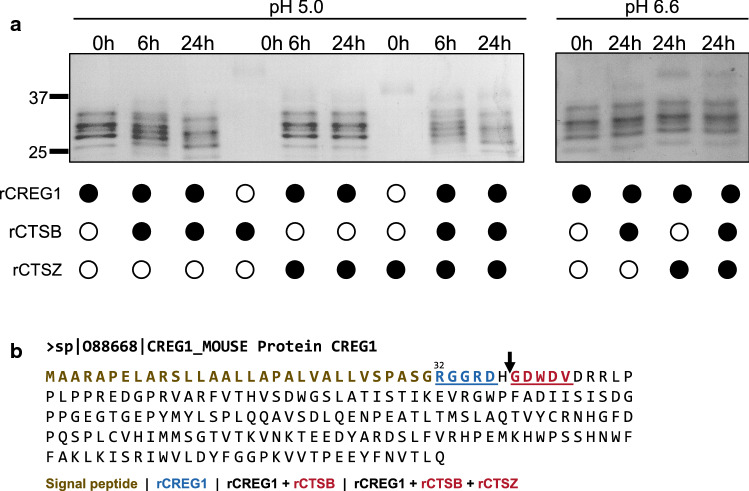
CREG1 is partially processed by CTSB generating a neo N-termini. **a** Partial processing of rCREG1 (2 µg) is observed by incubation with rCTSB (200 ng) after 6 h and increased after 24 h at pH 5.0. Incubation of rCREG1 with rCTSZ does not have significant changes; whereas, incubation of rCREG1 with both rCTSB and rCTSZ leads to a moderate increase in the processing at 6 and 24 h. At pH 6.6, no changes are observed with neither rCTSB, rCTSZ, nor with both CTSB and CTSZ. **b** N-terminal sequencing (Edman degradation) done with samples treated or not with rCTSB or rCTSB and rCTSZ after 24 h identified a potential CTSB cleavage site (Arrow H_37_–G_38_ bond). The sequence R_32_GGRD (blue) was identified in non-treated rCREG1 and the same neo N-termini GDWDV (red) was identified in samples treated with rCTSB or rCTSB and rCTSZ. Signal peptide (dark yellow); *rCREG1* recombinant murine CREG1, *rCTSB* recombinant murine CTSB, *rCTSZ* recombinant murine CTSZ

So far, we established an inverse relationship of cathepsin protease and CREG1 levels merely using mice or cells with constitutive, i.e., permanent, knock-out or overexpression of the protease. Therefore, we next aimed to manipulate the proteases more dynamically by protease inhibition (Fig. 5a) and inducible expression systems (Fig. 5b). CREG1 in CCM was increased upon treatment of PyMT cells with the cysteine cathepsin inhibitor E64d (Fig. 5a). In these conditions, CREG1 levels were similar to CCM of cells lacking *Ctsb*. Using a *Ctsb*^*−/−*^ PyMT cell line provisioned with a doxycycline-inducible system for expression of human CTSB [19], we found reduced levels of extracellular CREG1 upon CTSB induction (Fig. 5b). To gain insight into the cathepsin-dependent regulation of CREG1, we assessed its mRNA levels by quantitative real-time PCR. The co-culture system employed in the proteomic study was evaluated and no differences in the CREG1 mRNA level could be observed (Fig. 5c). Moreover, neither inhibition of cysteine cathepsins with E64d nor with the CTSB specific inhibitor CA-074 led to changes in CREG1 mRNA levels (Fig. 5d). As the results suggest a posttranscriptional regulation of CREG1 by the cathepsins, we next determined whether CREG1 would be a direct substrate for CTSB and/or CTSZ. To test this, mouse recombinant CREG1 was incubated with active recombinant mouse CTSB and/or CTSZ for 6 and 24 h at different pHs which would represent the intralysosomal compartment and extracellular space in the cancer setting (Fig. 6a). At pH 5.0, a partial CREG1 cleavage by CTSB was found; while, CTSZ alone did not noticeably process CREG1. Notably, the addition of CTSZ to CTSB moderately increased CREG1 degradation. Thus, CTSB is able to degrade CREG1 alone and in combination with CTSZ. In assays done at pH 6.6, no processing was observed in CREG1 upon addition of neither nor combination of enzymes, implying that the processing occurs in intracellular acidic vesicles, either located in the secretory pathway or in secretory lysosomes [67–69].

To determine the cleavage site of the proteases, recombinant CREG1 samples treated with CTSB or with both CTSB and CTSZ at pH 5.0 for 24 h were subject to N-terminal sequencing (Edman degradation) (Fig. 6b). In the untreated CREG1, the sequence RGGRD was obtained, which corresponds with the N-termini after the signal peptide (Fig. 6b, blue sequence). Incubation of CREG1 with CTSB as well as with both CTSB and CTSZ led to the same neo N-termini sequence GDWDV (Fig. 6b, red sequence) which lacks the first six amino acids at the N-termini in comparison to the untreated sample. Thus, R_32_GGRDH_37_^↓^G_38_DWDV (H_37_–G_38_ bond) represents a novel cleavage site which would correspond with the endopeptidase activity of CTSB, which is also known to occur near the N-terminus of proteins such as trypsinogen [14, 70].

### Impact of CREG1 on breast cancer cell growth and motility in 2D and 3D cell culture

**Fig. 7:**
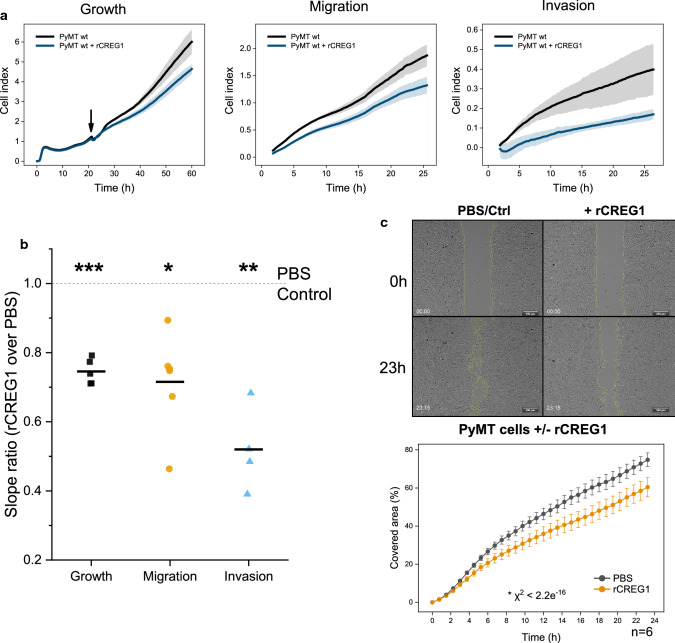
Extracellular rCREG1 can reduce cell growth, migration, and invasiveness of PyMT cells. **a** PyMT cells treated with rCREG1 (400 nM) show reduced cell growth, migration, and invasiveness in real-time cell monitoring with an xCELLigence system. An effect observed representatively in the generated impedance curve (**a**; upper panel) and **b** quantitively by the slope ratio when compared to PBS-treated control (dashed line). **c** Closure of the gap experiments with live-cell imaging show reduced closure area in PyMT cells treated with rCREG1 (400 nM) when compared to PBS-treated control PyMT cells. ***p value ≤ 0.001; **p value ≤ 0.01; *p value ≤ 0.05; *Chi-square of linear mixed-effects model. *wt* wild type, *rCREG1* recombinant murine CREG1

As CREG1 is degraded by cathepsin proteases, which have been shown to promote tumor progression in various genetic mouse models of human cancer including the MMTV-PyMT breast cancer model, we next addressed the role of CREG1 in growth and motility of PyMT breast cancer cells. After overnight incubation of PyMT cells, we added 400-nM recombinant CREG1 (rCREG1) [48] and monitored their growth in real time for at least additional 24 h using an xCELLigence™ (Acea Biosciences) device (Fig. 7a). Treatment with rCREG1 resulted in a significantly and consistently reduced slope, corresponding to a reduction in cell growth of around 30% when compared to control (Fig. 7a, left panel). Next, migration through a porous membrane and invasion through a Cultrex^®^ basement membrane extract towards a chemoattractant gradient (3% FCS) were measured. Consistently, a less steep slope (~ 20%) was observed in migration monitoring of cells treated with rCREG1 (Fig. 7a, middle panel). A more pronounced effect was detected on invasion, which was almost 50% reduced upon rCREG1 addition (Fig. 7a, right panel). The analysis of several biological replicates revealed statistical significance of these findings (Fig. 7b). In an alternative approach, the effect of rCREG1 on the migration of PyMT cells was evaluated in a gap-closure assay. For this PyMT cells were seeded flanking a silicone insert (ibidi GmBH). After overnight growth, insert removal created a defined 500-µm gap and media was replaced to 3% DMEM with or without 400-nM rCREG1. Subsequent live-cell imaging (JuLI™ Stage) proved a slower gap closure in the rCREG1-treated cell cultures (Fig. 7c).

**Fig. 8:**
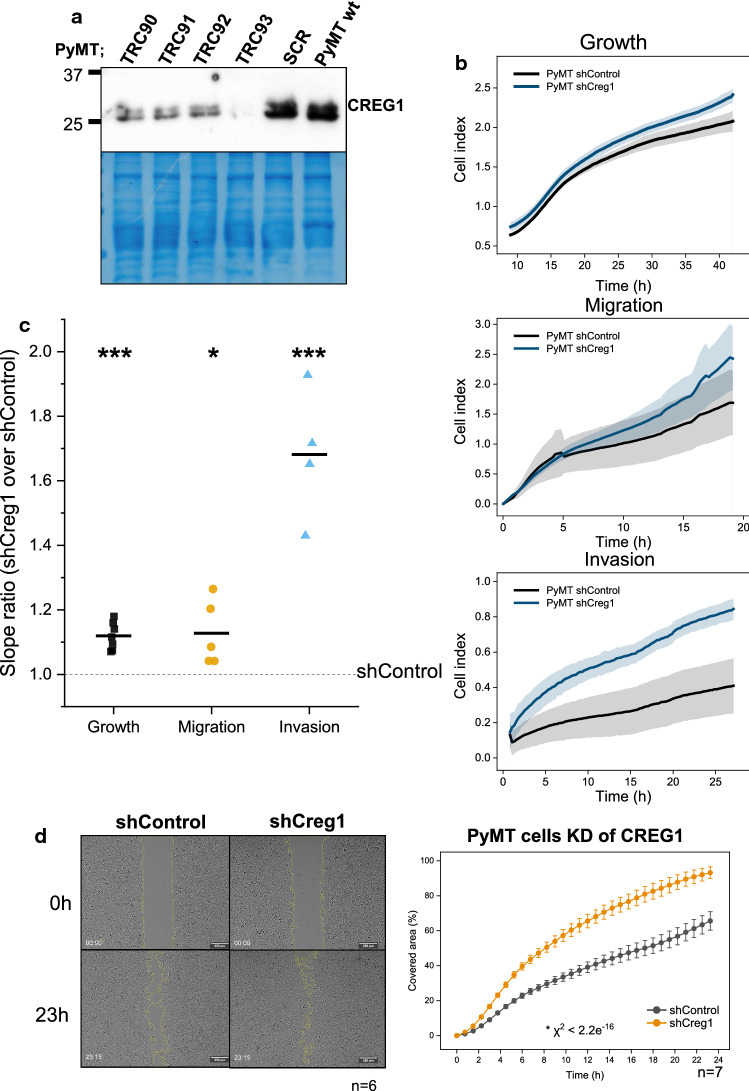
Reduced expression of CREG1 can increase cell growth, migration, and invasiveness in PyMT cells. **a** A PyMT cell-line with reduced *Creg1* expression (shCreg1) was generated by RNAi-mediated *Creg1* silencing, obtaining an important reduction with the shRNA named TRC93 and a control cell line (shControl) with the shRNA named SCR. **b** Real-time cell monitoring with an xCELLigence device shows increased cell growth, migration, and invasiveness, representatively observed in the generated impedance curves and **c** quantitively significant using the slope ratio when compared to a shControl PyMT cell line (dashed line). **d** Closure of the gap experiments with live-cell imaging shows increased closure area in shCreg1 PyMT cells when compared to shControl PyMT cells. ***p value ≤ 0.001; *p value ≤ 0.05. *wt* wild type, *shControl* PyMT cell line with control shRNA, *shCreg1* PyMT cell line with reduced *Creg1* expression

Complementary to the CREG1 gain-of-function experiments using rCREG1, we next assessed if reducing CREG1 in PyMT cells would lead to the opposite effect. For this, four different anti-*Creg1*-shRNAs, as well as a control shRNA (non-target shRNA control plasmid, SCR), were transfected into the parental PyMT cell line. Out of these, one shRNA (TRC93), named hereafter shCreg1, achieved a substantial reduction of CREG1 at the protein level (Fig. 8a), with no apparent difference in comparison to the control shRNA and the parental cell line (Supplementary File 2, Fig. 1A). Comparing the *Creg1* knock-down and the shControl cells in xCELLigence real-time assays, we found a slight, but significant 12% increase in the growth of shCreg1 cells (Fig. 8b upper right panel, c). Alongside, migration of these cells showed a similar increase compared to the shControl (Fig. 8b middle right panel, c). Furthermore, we found a consistently higher invasion of shCreg1 relative to shControl cells (Fig. 8b lower right panel, c). Likewise, gap closure by shCREG1 cells was faster than by the shControl (Fig. 8d). Additionally, and to discard single-cell clone biases, we generated a second set of cell lines using the same anti-sense targeting *Creg1* and control shRNA, obtaining a successful independent knock-down. Increased growth, migration, and invasion were observed as for the original knock-down cells (Supplementary File 2, Fig. 2A).

**Fig. 9:**
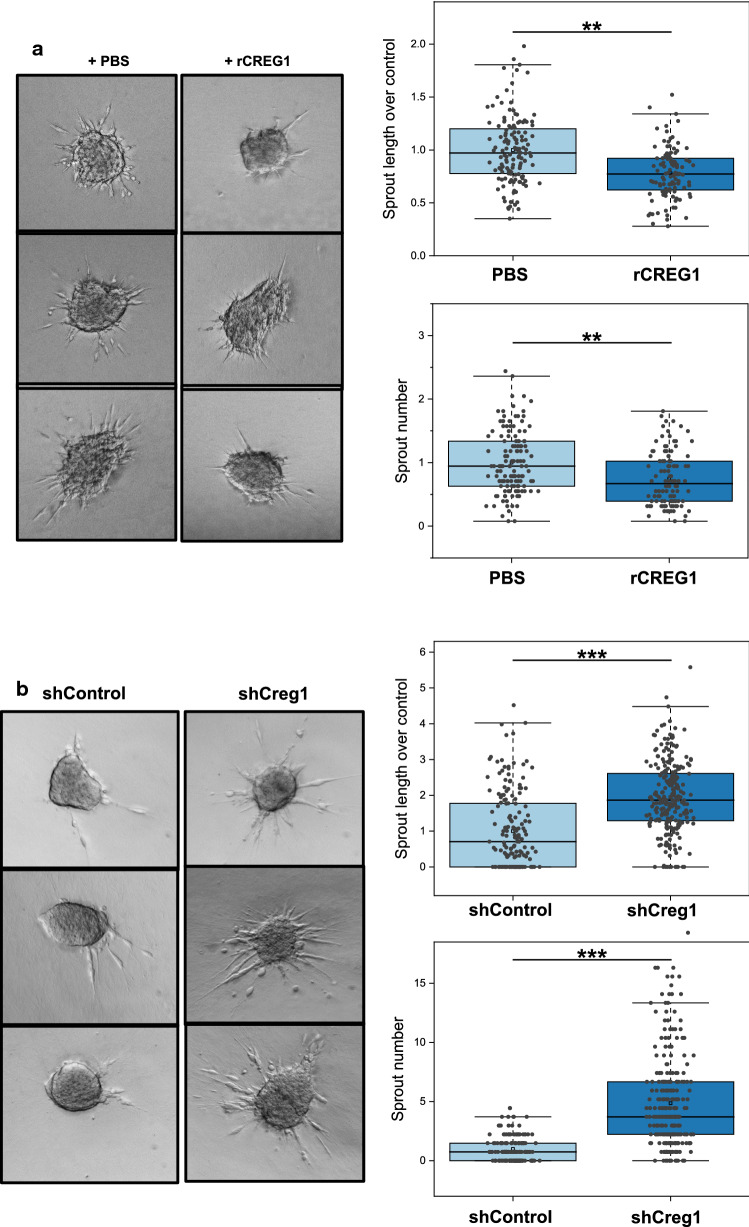
Migration and invasiveness of PyMT cells can be modulated by CREG1. **a** PyMT cell 3D sphere cultures were generated in a methylcellulose/collagen I matrix show reduced sprout number and length when treated with rCREG1 (400 nM) for 24 h, representatively observed in spheroid images (upper left) and quantitively (upper right). **b** 3D spheres generated with PyMT cells with reduced *Creg1* expression (shCreg1) show increased sprout number generation and length, detected representatively by spheroid images (lower left) and quantitively (lower right). **p value ≤ 0.01; ***p value ≤ 0.001. *rCREG1* recombinant murine CREG1, *shControl* PyMT cell line with control shRNA, *shCreg1* PyMT cell line with reduced *Creg1* expression

To further assess the effect of CREG1 in a three-dimensional tissue context, we generated PyMT cell 3D-sphere cultures in methylcellulose/collagen I matrix [19]. These spheres form multicellular sprouts invading the surrounding matrix (Fig. 9). PyMT cells with 400-nM rCREG1 added to the 3D culture showed significantly fewer and shorter sprouts (Fig. 9a). In line with our previous findings, depletion of CREG1 in shCREG1 cells resulted in significantly increased sprout formation (Fig. 9b).

Taken together, the results provide consistent evidence for an inverse association of CREG1 protein levels to growth and invasion properties of PyMT breast cancer cells, i.e. a high CREG1 level suppresses the malignant cell behavior, while low CREG1 promotes it.

### Macrophage-derived CREG1 impairs invasiveness of PyMT spheroids

**Fig. 10:**
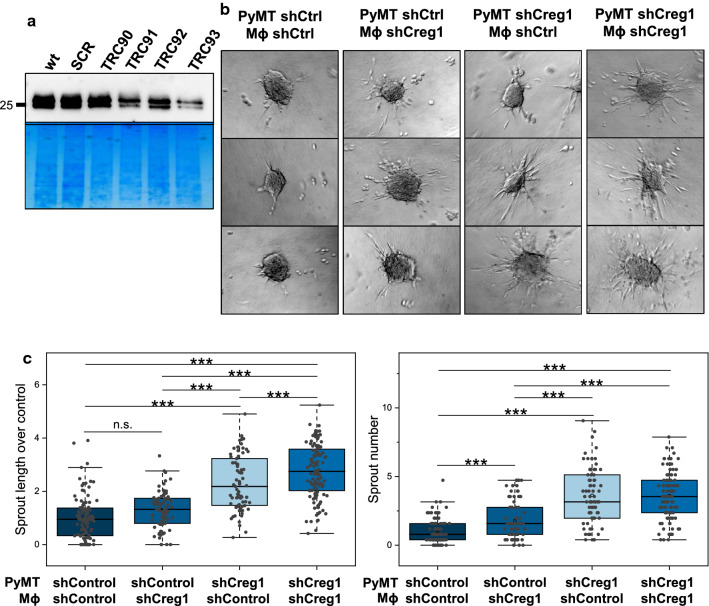
Macrophage-derived CREG1 halts the migration and invasiveness of PyMT spheroids in 3D cultures. **a** A macrophage cell line with reduced *Creg1* expression was generated by RNAi-mediated CREG1 silencing, obtaining an important reduction with the shRNA named TRC93 and a control cell line (shControl) with the shRNA named SCR. **b** Co-culture of PyMT cell spheroids in a methylcellulose/collagen I matrix with reduced *Creg1* expression (shCreg1) or not (shControl) with Mϕ knock-down or not for *Creg1*. **c** Co-cultures with control PyMT cells and *Creg1* knock-down Mϕ show increased sprout number. Moreover, reduce levels of *Creg1* in PyMT cells in the presence of shControl Mϕ show increased sprout length and number; whereas, co-cultures with *Creg1* knockdown PyMT cells and Mϕ show a moderate increase in sprout number. ***p value ≤ 0.001. *n.s.* non-significant change, *wt* wild type, *Mϕ* macrophage, *SCR* shControl, *ShCtrl/shControl* PyMT cell line with control shRNA, *shCreg1* PyMT cell line with reduced *Creg1* expression

Our initial proteomic findings indicated Mɸ as a major source of CREG1. To investigate the action of Mɸ-derived CREG1, we next established a shRNA-mediated *Creg1* knock-down in a mouse Mɸ cell line (Fig. 10a). The macrophage morphology remained unchanged upon *Creg1* knock-down (Supplementary File 2, Fig. 1B). Our previous work has established that co-culture of Mɸ and PyMT spheroids promotes the formation of invasive cancer strands into a collagen I matrix [19]. Here, we determined invasive strand formation depending on *Creg1* knock-down in tumor cells, in Mɸ or in both cell types (Fig. 10b, c). As observed for PyMT spheroids without macrophage addition (Fig. 9b), shCREG1 knock-down PyMT cells in the presence of shControl Mɸ resulted in a larger and increased number of sprouts. On the other hand, shCreg1 knock-down Mɸ led to significantly more sprouts when co-cultivated with shControl PyMT cells (Fig. 10c). Combining *Creg1* knock-down cancer cells and Mɸ did not boost invasive sprout numbers but caused a small but significant increase in sprout length. These experiments support the idea that Mɸ-derived CREG1 can at least partially suppress invasive behaviors of cancer cells in complex 3D-environments.

### Reduced CREG1 expression promotes tumor progression in vivo

**Fig. 11:**
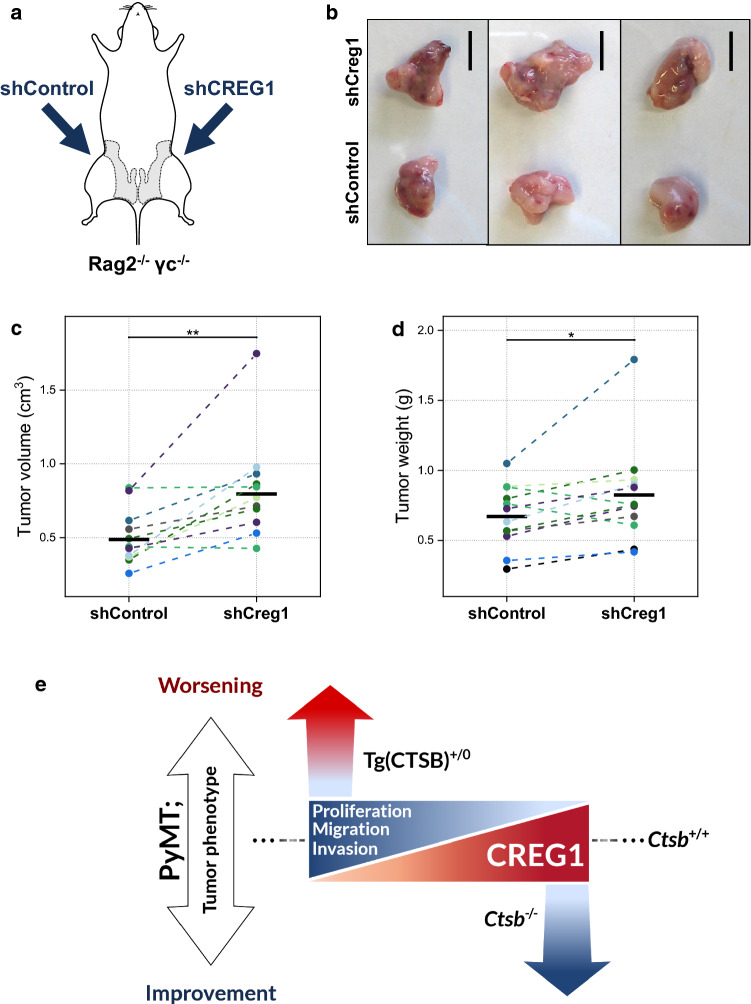
Reduced *Creg1* expression leads to tumor progression in vivo. **a** PyMT cells with reduced expression of *Creg1* (shCreg1) or shControl PyMT cells were orthotopically transplanted concomitantly into the mammary fat pad of Rag2^−/−^ γc^−/−^ immunosuppressed mice, employing each mouse as its own control. **b** After 4 weeks, tumors generated by shCreg1 PyMT cells had a larger aspect. **c** Excised tumor masses revealed larger volume and **d** increased weight when pair-wise compared to tumor masses formed by shControl PyMT cells. **e** Proposed model on how CREG1 could act in the context of the contrasting tumor phenotype observed upon differential CTSB expression in the MMTV-PyMT mouse model of breast cancer. Transgenic expression of human CTSB leads to increased proliferation, migration, and invasion and lower CREG1 levels; whereas, ablation or inhibition of CTSB leads to the opposite effects, while with CREG1 increased abundance. Thus, CREG1 may be an important player in the crosstalk interactions at the tumor-microenvironment which lead to an opposing tumor phenotype seen with differential CTSB expression. Scale bar: 1 cm. **p value ≤ 0.01; *p value ≤ 0.05. *shControl* PyMT cell line with control shRNA, *shCreg1* PyMT cell line with reduced *Creg1* expression

Lastly, to evaluate the influence of CREG1 on tumor progression in vivo, shCreg1 PyMT cells were orthotopically transplanted into the mammary fat pad of Rag2^−/−^ γc^−/−^ immunosuppressed mice [22, 23]. Identical numbers of shControl cells were transplanted in the contralateral mammary fat pad, thereby creating a matched-pairs experimental design (Fig. 11a). After transplantation, mice were followed up and tumors were harvested after 4 weeks. At this time point, tumors stemming from shCreg1 PyMT cells were larger in aspect (Fig. 11b). This was quantitatively supported by a significantly increased tumor volume (Fig. 11c) as well as a significantly elevated tumor weight as compared to the matched shControl PyMT tumors (Fig. 11d). This experiment is the first evidence for an impact of CREG1 expression levels on tumor growth in vivo.

## Discussion

Tumor–stroma interactions are decisive for carcinogenesis [71, 72]. CTSB, a lysosomal protease that is also secreted into the stroma, is an important player in these interactions due to its proteolytic ability to shape the extracellular matrix [1, 2, 12]. In the MMTV-PyMT model of metastasizing breast cancer, the transgenic expression of human CTSB is associated with faster tumor growth, enlarged tumor size, increased number and size of metastasis, and higher grade of malignancy [16, 19]. In contrast, CTSB deletion leads to delayed tumor onset, slower growth rate, and reduced metastasis volume [13]. Moreover, simultaneous ablation of CTSB and CTSZ leads to synergistic anti-tumoral effects with delayed tumor onset, reduced number and size of metastases, and improved histopathological scores [21]. Yet, the cathepsin-dependent molecular mediators of these phenotypes are not well understood. To explain the cancer phenotype of the cathepsin knock-outs, one must search either for tumor-promoting proteins activated by cathepsin proteases or tumor-suppressing proteins being inactivated by proteolytic cleavage [5]. Here we screened for such potential cathepsin-dependent mediators by mass-spectrometry-based proteomics in vitro using the cell-conditioned media of PyMT cell/macrophage co-cultures as well as in vivo using the tumor interstitial fluid of MMTV-PyMT tumors. Importantly, we identified the glycoprotein CREG1 to have a reduced abundance upon the transgenic expression of human CTSB; while, its levels increased when CTSB or CTSB/CTSZ were absent or when CTSB was inhibited. Interestingly, CREG1 is a secreted and also lysosomal protein with previously reported tumor-suppressor-like functions [73–75]. Both CREG1 and CTSB are lysosomal proteins, subject to lysosomal sorting, and therefore potentially prone to lysosomal exocytosis in the tumor context [76]; thus, both proteins are spatiotemporally located in the same space, strengthening the idea that such an interaction occurs in vivo. Hence, CREG1 was a strong candidate to explain the ameliorated phenotype of CTSB knock-out in the MMTV-PyMT breast cancer model (Fig. 11e).

The results of the proteome analysis on CREG1 were corroborated by Western blot and immunohistochemistry demonstrating the increased abundance of CREG1 both in vitro and in vivo. Additionally, inhibition or overexpression of CTSB led to increased or reduced levels of CREG1, respectively. The increase of CREG1 was not associated with changes at the mRNA level, thereby reinforcing the idea of CREG1 being a CTSB substrate. Along those lines, we could validate published evidence on the proteolytic cleavage of CREG1 by CTSB [77]. In our in vitro experiments, we observed a partial CTSB-mediated cleavage of CREG1, which was further enhanced by the addition of CTSZ. Moreover, we found a CTSB cleavage site in CREG1 removing the first six amino acids of the N-terminus. There are other examples for such endoproteolytic cleavage by CTSB in the processing of hormones, zymogens, or apoptotic factors [70, 78–80]. Maybe most impressive is the CTSB-mediated removal of the N-terminal octapeptide of trypsinogen. Removal of this “trypsinogen activation peptide” in acinar cells of the pancreas results in premature trypsinogen activation—one of the critical steps in the pathogenesis of pancreatitis [70]. We interpret our findings on CREG1 as sequential proteolysis with CTSB doing first an endoproteolytic cleavage, which would then allow the CTSZ carboxypeptidase activity to further process. Nonetheless, CTSB/CTSZ-mediated proteolysis of CREG1 in vitro did not lead to its absolute disappearance, suggesting the involvement of further proteases for complete degradation of CREG1 in vivo. A common feature of proteases is their high degree of functional interconnection thereby forming proteolytic cascades, proteolytic systems, and the so-called protease web [81, 82]. Notably, the system executing the degradation of CREG1 might include other members of the cathepsin family. Kowalewski-Nimmerfall et al. observed processing of the *Drosophila melanogaster* homolog CREG1 when exposed to cathepsin l as well as to CTSB [77].

Previous reports have shown that CREG1 expression was associated with reduced proliferation and enhanced differentiation in several types of cells [73, 83–87]. In contrast, reduced expression resulted in opposite effects [64, 84, 86–88]. In addition to proliferation and differentiation, CREG1 also reduced cell migration [87, 89, 90]. Here, we show CREG1 gain- and loss-of-function experiments in the context of the MMTV-PyMT breast cancer model. We established an inhibitory effect of CREG1 on invasion in both 2D and 3D cultures, which is of great interest in the oncologic setting. We also provide first in vivo evidence for tumor-suppressing functions of CREG1 in orthotopic transplantation of CREG silenced cancer cells into mouse mammary fat pad. We observed that CREG1 production is substantial in Mɸ, as is the case for cathepsins [6, 7, 9]. In vivo Mɸ might well be the main source for CREG1. Further experiments were carried out by the addition of recombinant CREG1 to mimic macrophage secreted CREG1 in the tumor microenvironment. However, CREG1 seems to have a tumor cell-autonomous effect, as silencing CREG1 in PyMT cells led to increased proliferation, migration, and invasion. Nonetheless, we also found a small but significant increase of invasive strand formation of 3D PyMT cell spheres co-cultivated with CREG1-silenced Mɸ. Hitherto, one might envision that both Mɸ and tumor cells secrete CREG1, which then contributes to tumor control. Often, cancer cells and tumor-associated Mɸ overexpress cathepsins, such as CTSB and CTSZ [2, 17, 18]. CREG1 appears to be one of their important substrates whose cleavage supports tumor growth. Conversely, cathepsin inhibition or silencing spares CREG1 from degradation and enables CREG1-mediated attenuation of tumor progression.

What is known about the molecular mechanisms by which CREG1 exerts its effects on cell growth? Recent reports show that CREG1 hampers diet-induced obesity and hepatic steatosis in mice, and its deletion resulted in insulin resistance [91–94]. These CREG1 functions might be associated with the JNK pathway [92] and/or due to its stimulation of expression of the uncoupling protein 1 [94]. CREG1 can also bind to the retinoid X receptor α, which in turn can interact with the thyroid hormone receptor, thereby promoting brown adipogenesis [93]. Although these late reports associate CREG1 functions with downstream signaling pathways, its tumor-suppressor-like functions might reside in its binding to the cation-independent mannose-6-phosphate insulin-like growth factor 2 receptor (M6P/IGF2R) [83]. The M6P/IGF2R is a multiple ligand-binding cell surface receptor, with reported tumor suppressor properties in several cancer entities [95]. One of its main functions is the sorting of lysosomal proteins and the internalization of extracellular growth factors, like IGF2, for lysosomal degradation, thus acting as a tumor-suppressor receptor [95, 96]. The two opposing glycosylation sites that CREG1 has [66, 97–99], not only facilitate its binding to M6P/IG2FR, but also deletion of the receptor abrogates CREG1-mediated growth inhibition [83]. Additional reports have provided further evidence for CREG1 functions through the M6P/IGF2 receptor. CREG1-silencing in fibroblasts led to growth promotion, which was reduced by the addition of recombinant CREG1; whereas, this effect was abrogated by the addition of an M6P/IGF2R neutralizing antibody [86]. Moreover, *Creg1* knock-down led to diffuse M6P/IGF2R cellular localization, which was reverted to a more focal distribution by addition of recombinant CREG1. In a follow-up study, CREG1 effects on migration in human vascular smooth muscle cells were reported to be mediated through M6P/IGF2R [89]. Altogether, these studies provided experimental evidence that the CREG1-mediated inhibition of cell proliferation and migration is likely to be achieved by CREG1-mediated regulation of the M6P/IGF2R sorting, including the secretion, re-uptake, and lysosomal targeting of IGF2 [95, 100].

In conclusion, we were able to establish CTSB as a key determinant of CREG1-mediated tumor growth suppression. The CTSB–CREG1 axis explains at least in part the frequently reported tumor attenuation upon CTSB knock-out or inhibition in MMTV-PyMT breast cancers and other cancer models (Fig. 11e). Consequently, we suggest that pharmacological targeting the enzyme CTSB represents a non-genetic tool to change the abundance of the otherwise hard to target glycoprotein CREG1 in future functional studies.

### Electronic supplementary material

Below is the link to the electronic supplementary material.

Supplementary file1 (XLSX 332 kb)

Supplementary file2 (PDF 3347 kb)

Supplementary file3 (XLSX 2161 kb)

## Data Availability

The LC–MS/MS proteomics data have been deposited to the ProteomeXchange Consortium via the PRIDE partner repository with the dataset identifier PXD015209.

## Abbreviations

CCM: Cell-conditioned medium
CA-074: (l-3-trans-(propylcarbamyl)oxirane-2-carbonyl)-l-isoleucyl-l-proline
CTSB: Cathepsin B
CTSZ: Cathepsin Z
CREG1: Cellular Repressor of E1A Stimulated Genes 1
E64: Trans-epoxysuccinyl-l-leucylamido(4-guanidino)butane
E64d: (2S,3S)-trans-epoxysuccinyl-l-leucylamido-3-methylbutane ethyl ester
FC: Fold change
LC–MS/MS: Liquid chromatography–tandem mass spectrometry
Mϕ: Macrophage
M6P/IGF2R: Cation-independent mannose-6-phosphate Insulin-like growth factor 2 receptor
MMTV: Mouse mammary tumor virus
PyMT: Polyoma middle T antigen
shRNA: Short hairpin RNA
SCX: Strong cation exchange
TAM: Tumor-associated macrophage
TIF: Tumor interstitial fluid
