# Supplementary material for: The secreted inhibitor of invasive cell growth CREG1 is negatively regulated by cathepsin proteases
Source: Cell Mol Life Sci. 2020 May 8;78(2):733–55. doi: 10.1007/s00018-020-03528-5 (PMC7873128; doi:10.1007/s00018-020-03528-5)
Supplement: Supplementary file 2 — Supplementary file2 (PDF 3347 kb) [file 18_2020_3528_MOESM2_ESM.pdf]

# Supplement Figure 1

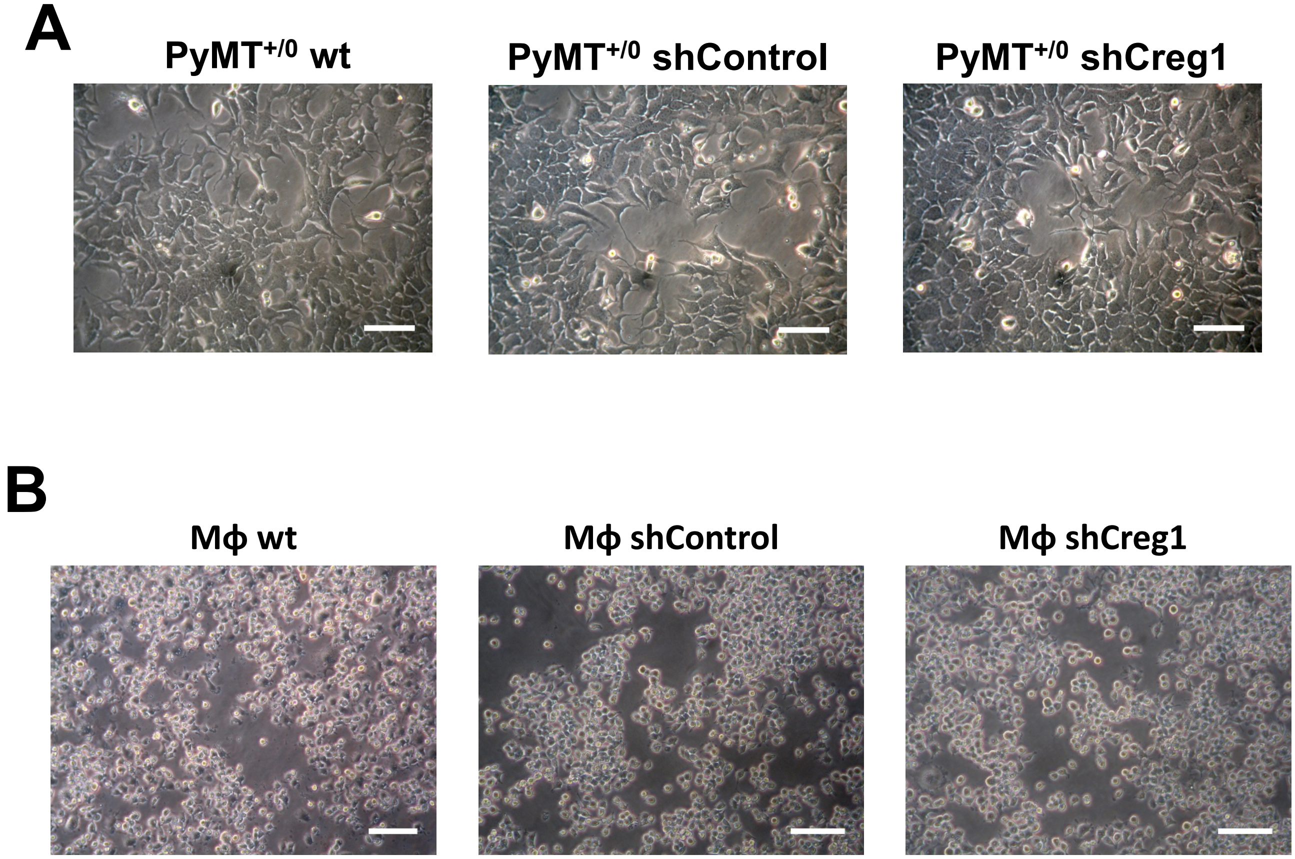


**Supplement Figure 1. RNAi-mediated silencing of CREG1**
*Creg1* expression was reduced by RNAi-mediated silencing using shRNAs in PyMT cells and Macrophages. A good expression reduction was observed with one shRNA (TRC93; shCreg1). (A) No apparent morphological differences are observed with the shRNA targeting *Creg1* (shCreg1) nor with a control shRNA (shControl) in (A) PyMT cells nor in (B) macrophages. Scale bar: 100 µm. Mϕ: Macrophage

# Supplement Figure 2

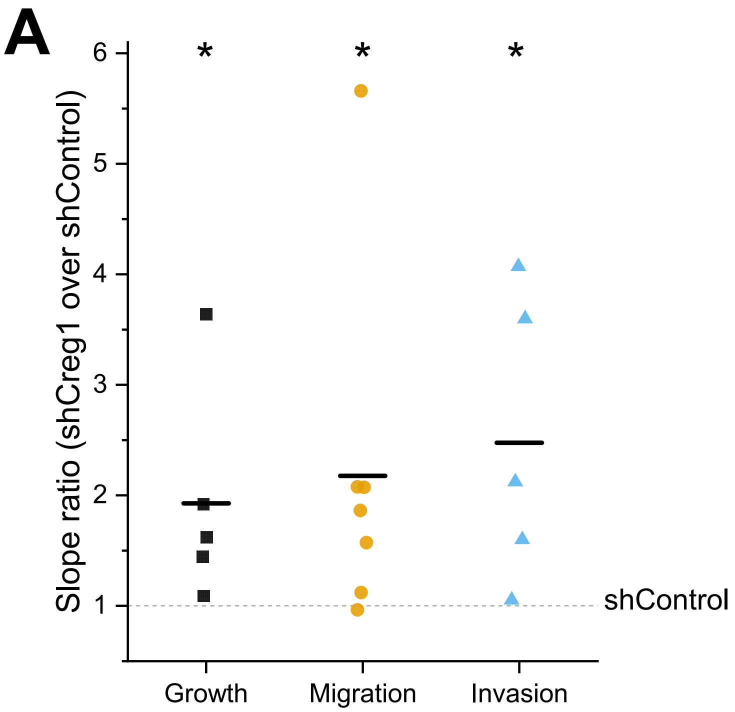


**Supplement Figure 2. Reduced expression of CREG1 can increase cell growth, migration, and invasiveness in PyMT$^{+/0}$ cells**

(A) Expression of *Creg1* was reduced in a second PyMT cell-line using the same shRNAs and obtaining a reduction with the shRNA named TRC93 (shCreg1). An shRNA control cell line was generated as well with a control shRNA (shcontrol). Real-time cell monitoring with an xCELLigence device shows increased cell growth, migration, and invasiveness. * p-value ≤ 0.05. shControl: PyMT cell line with control shRNA; shCreg1: PyMT cell line with reduced *Creg1* expression
